# Limited Therapeutic Time Windows of Mild-to-Moderate Hypothermia in a Focal Ischemia Model in Rat

**DOI:** 10.4061/2011/131834

**Published:** 2011-08-16

**Authors:** Heng Zhao, Gary Steinberg

**Affiliations:** ^1^Department of Neurosurgery, Stanford University School of Medicine, Stanford, CA 94305-5327, USA; ^2^Stanford Stroke Center, Stanford University School of Medicine, Stanford, CA 94305-5327, USA

## Abstract

Although many studies have shown the great potential of induced hypothermia in stroke treatment, we recognize that there are limitations to the protective effects of hypothermia even in the laboratory. Here, we review our experiments on the protective effects of mild-to-moderate hypothermia in rats. Focal ischemia was induced by bilateral common carotid artery (CCA) occlusion for 1 to 2 hours combined with permanent or transient middle cerebral artery (MCA) occlusion. We compared the effects of mild (33°C) and moderate (30°C) hypothermia, evaluated therapeutic time windows, and studied the underlying mechanisms. On review, our findings revealed that the protective effects of induced mild hypothermia (33°C) were limited, and the therapeutic time window of even moderate hypothermia (30°C) was very short in our specific models, although this limitation might be due to the relatively brief periods of hypothermia used. In addition, we found that hypothermia reduced brain injury by preserving Akt activity, PTEN phosphorylation and **ε**PKC activity, while inhibiting ROS production, and **δ**PKC activity.

## 1. Introduction

One of the gold standards of neuroprotectants against stroke in animal experiments [1, 2] induced mild (33 to 36°C) to moderate (28 to 32°C) hypothermia has been the focus of several clinical trials for the treatment of cerebral ischemia. In the past decade, prospective randomized controlled studies have demonstrated that induced hypothermia improves neurological function in patients suffering cardiac arrest from ventricular fibrillation [3] and reduces risk of death or disability in neonates following hypoxic-ischemic encephalopathy [4, 5]. However, the clinical translation of hypothermia for acute stroke treatment is still in its early stages. Many barriers remain, including onset time, duration, and depth of hypothermia [6]. 

In the process of extrapolating animal studies to human patients, significant gaps exist even between the design of laboratory experiments and clinical trials. For instance, many previous animal models used complete reperfusion [7–9], while most stroke patients suffer from permanent cerebral artery occlusion [10, 11]. Even with t-PA treatment, slightly less than one third of patients achieve complete reperfusion, one-third achieve partial reperfusion, and in the rest reperfusion is absent [11, 12]. Therefore, the ability to select animal stroke models that properly mimic clinical stroke is a critical step in evaluating the protective effects of induced hypothermia. 

Our laboratories have studied the protective effects of mild-to-moderate hypothermia for nearly two decades [6, 13–17]. Our recent hypothermia studies use a focal ischemic model with partial reperfusion in rats [16, 18, 19]; a model which is less frequently used in other laboratories. In this model, stroke is induced by bilateral common carotid artery (CCA) occlusion combined with permanent distal middle cerebral artery (MCA) occlusion [16, 20–22]. The bilateral CCAs are reopened 1 to 2 hours later while the distal MCA remains occluded [16, 19, 23, 24]. This technique therefore allows partial reperfusion [25, 26]. As discussed above, this model mimics many stroke patients who receive partial reperfusion, with or without t-PA treatment. However, to compare the protective effects of hypothermia in focal ischemia with partial reperfusion and complete reperfusion, we also used a model with transient three-vessel (bilateral CCAs and distal MCA) occlusion [18].

Several excellent articles have reviewed the protective effects of hypothermia as function of onset time, duration, and depth of hypothermia, as well as its underlying protective mechanisms [27–30]. Particularly, van der Worp et al. have comprehensively reviewed past hypothermic studies [29], which either used temporary or permanent occlusion models. However, the protective affects of hypothermia in stroke models using partial reperfusion as described above have received significantly less attention. Therefore, this paper focuses mainly on our studies of the past several years on therapeutic time windows and the unique model of partial reperfusion.

## 2. Intraischemic Moderate Hypothermia Offers Strong and Long-Term Protection in a Focal Ischemic Model with Partial Reperfusion

**Figure 1:**
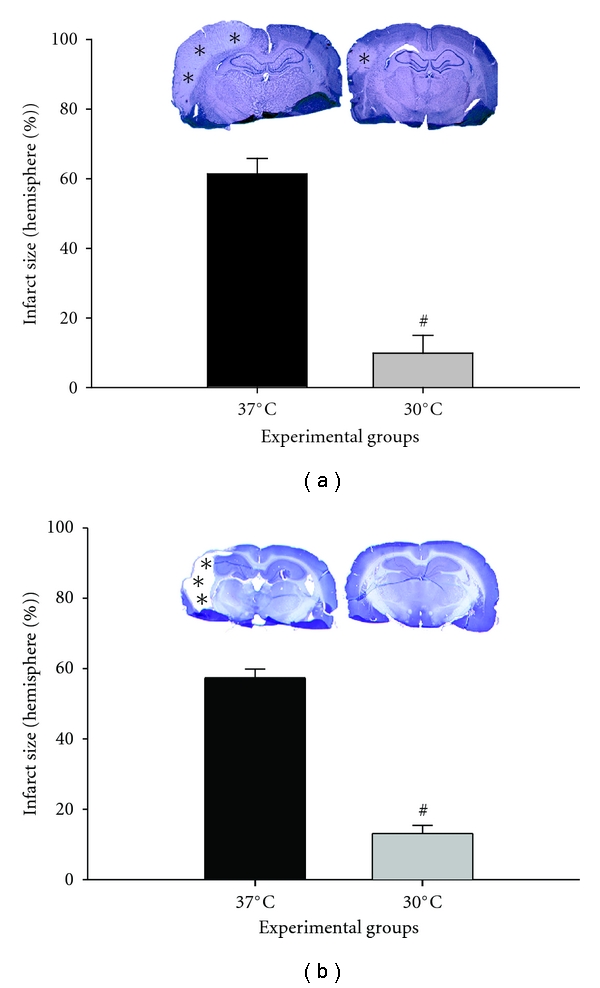
(Revised from [16]). Intraischemic moderate hypothermia (30°C) reduces infarct size in a focal ischemia with partial reperfusion. Focal ischemia was induced by 1 h of bilateral CCA occlusion and permanent dMCAo. Body core temperature was lowered to 30°C 10 min before stroke onset by spraying 70% alcohol on the rat body. (a) The upper panel shows representative infarcts stained with cresyl violet from rats euthanized 2 d after stroke. The pale area with asterisks represents the infarct region. Normothermic ischemia damaged the cortex ipsilateral to the occluded MCA, whereas hypothermia spared all or most of the injured cortex. Only a small lesion was observed in the presented section from a hypothermic rat. The bar graphs represent statistical analysis of infarct size 2 d after stroke. Two-way ANOVA (two factors, temperature and brain section level) was used to compare the effect of temperature on the infarct size at each level (data not shown) and on the mean of all 4 levels. Hypothermia (*n* = 7) reduced the mean infarct size by 80% compared with normothermia (*n* = 7; *P* = 0.001). (b) The upper panel shows representative sections stained with cresyl violet from animals surviving 2 months after stroke. Most of the cortex in the infracted hemisphere was lost in normothermic but not hypothermic rats. The lower panel of bar graphs shows infarct size 60 d after stroke. Hypothermia (*n* = 9) reduced infarct size 60 d after stroke compared with normothermia (*n* = 8; *P* = 0.001). # versus 37°C, *P* < 0.001.

In our first implementation of an ischemic model [16], we cauterized the distal MCA above the rhinal fissure and transiently occluded the bilateral CCAs for 1 hour. This model generates a well-delineated ischemic area limited to the cortex [20, 22]. Moderate hypothermia (30°C) monitored at the core body temperature was induced 10 minutes before ischemia onset and maintained for 1 hour after ischemia onset [16]. Although we did not directly monitor brain temperature, we previously observed a high correlation between rectal temperature and brain temperature in hypothermic rats [21]. We should add that because brain temperature in normothermic rats drops spontaneously during occlusion, core temperature may not accurately reflect brain temperature [2, 31]. Even so, we did not experimentally adjust any potential changes in brain temperature in order to minimize the introduction of possible artificial factors, which would likely exacerbate ischemic injury once the brain was heated. Our results showed that hypothermia reduced infarct size more than 80% compared with normothermia at 2 days after stroke (Figure 1(a)) [16]. Because some neuroprotectants offer transient protection, we also measured brain injury 2 months later and found similar protective effects at 60 days and 2 days (Figure 1(b)), suggesting that hypothermia decreases ischemic damage over the long term rather than merely delaying its emergence. This protective effect is further strengthened by the effects of hypothermia on behavioral deficits after stroke, which showed that hypothermia improved neurological functioning for up to 2 months [16]. 

**Figure 2:**
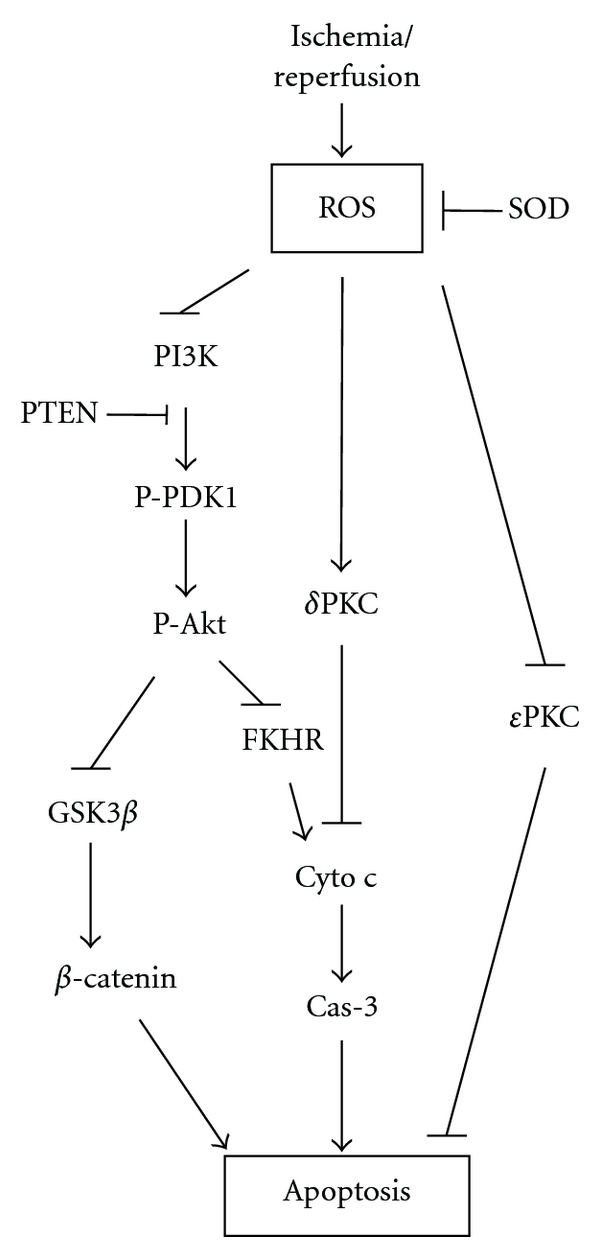
Diagram showing the major cascades that occur after stroke reviewed in this paper. AD: anoxic depolarization; AIF: apoptosis-inducing factor; BBB: blood brain barrier; CBF: cerebral blood flow; cyto c: cytochrome c; Fas L: Fas ligand; FKHR: Forkhead homologue in rhabdomyosarcoma; Glu: glutamate; GSK 3 *β*: glycogen synthase kinase 3*β*; MMP: matrix metalloprotease; NOS: nitric oxide synthesis; NO: nitric oxide; ONOO^−^: peroxynitrite; PI3K: phosphoinositide 3-kinase; PIP2: phosphatidyliositol-4,5-bisphosphate; PIP3: phosphatidyliositol-3,4,5-bisphosphate; PKC: protein kinase C; P-Akt: phosphorylated Akt; PTEN: phosphatase and tensin homologue deleted on chromosome 10; P-PDK1: phosphorylated phosphoinositide-dependent protein kinase-1; ROS: reactive oxygen species; RTK: receptor tyrosine kinase; VDCC: voltage-dependent calcium channel.

We then used this model to study the underlying protective mechanisms related to the PI3K/Akt cell signaling pathway [16]. The PI3K/Akt kinase pathway is known to promote neuron survival postischemia (reviewed by [32]) Figure 2. Akt activity is regulated by phosphorylation at Ser-473 and Thr-308 via upstream molecules, such as PDK1 and PTEN. While activated PDK1 phosphorylates Akt, activated PTEN dephosphorylates Akt. Activated Akt then blocks caspase/cytochrome c-mediated apoptosis by phosphorylating Akt substrates, such as FKHR and GSK3*β*. In our study, stroke resulted in transient increases in phosphorylated Akt (P-Akt) levels, but led to a reduction in phosphorylation levels of PTEN, PDK1, GSK3*β*, and FKHR [16]. However, in vitro Akt kinase assays showed that true Akt activity was decreased after stroke. Although hypothermia blocked the increase in P-Akt after stroke, it maintained true Akt activity. A functional role for this hypothermia-maintained activity is supported by the finding that the PI3K/Akt inhibitor, LY294004, enlarged infarct size in hypothermic animals. In addition, hypothermia attenuates a decrease in P-PTEN after stroke onset. Taken together, our results suggest that the PI3/Akt pathways play a critical role in the neuroprotection observed in intraischemic moderate hypothermia [16]. 

We also studied the potential roles of two critical components in the protein kinase C (PKC) pathway: *δ*PKC [24] and *ε*PKC [23]. *δ*PKC is a kinase strongly implicated in executing ischemic damage while *ε*PKC is neuroprotective [33]. We found that intraischemic hypothermia (30°C) blocks translocation of *δ*PKC to the mitochondria and nucleus and attenuates *δ*PKC cleavage [24], but it promotes *ε*PKC activity, as evidenced by increased *ε*PKC phosphorylation levels [23]. Therefore, our results suggest that both *δ*PKC and *ε*PKC may participate in the protective effects of intraischemic moderate hypothermia. 

## 3. Intraischemic Mild Hypothermia (33°C) Fails to Offer Protection in a More Severe Ischemic Model with Partial Reperfusion

**Figure 3:**
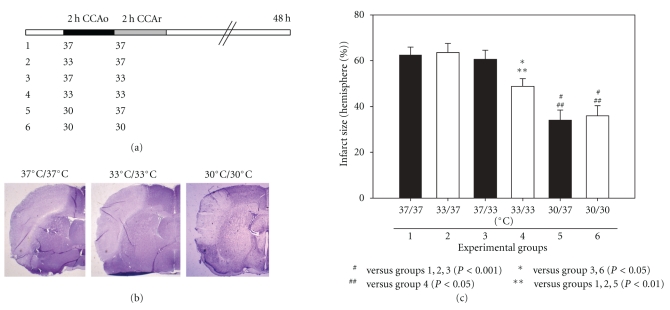
(Revised from [19]) (a) Protocols for surgery and temperature management. Six groups of rats were studied. The distal MCA was occluded permanently. The *black portion *of the bar represents bilateral CCA occlusion (CCAo) for 2 h, and the *gray portion *indicates 2 h of temperature management after CCA release (CCAr), including 30°C, 33°C, and 37°C. Rats were allowed to survive for 48 h after stroke. (b) Photographs of representative infarct sections after cerebral ischemia from groups 1, 4, and 6. Permanent distal MCA occlusion plus 2 h of bilateral CCA occlusion caused an infarct in the ipsilateral cortex of the occluded MCA (*left, *group 1). A coronal section from Level 2 is presented. Four hours of mild hypothermia (*center, *group 4) mildly decreased infarct size. When the temperature was reduced to 30°C robust protection was observed (*right, *group 6). (c) Bar graph showing that hypothermia reduces infarct size after stroke only under certain conditions. A mean infarct size for each group was calculated as the sum of all 4 levels for each animal divided by the number of animals in each group. The infarct size did not differ among groups 1 through 3. However, the infarct in group 4 was reduced about 22% relative to group 1. When the temperature was decreased to 30°C (group 5) robust protection was observed; an additional 2 h of hypothermia in group 6 did not further reduce infarct size.

In our second study we compared the protective effects of mild (33°C) and moderate hypothermia (30°C) [19] either transiently induced during or after CCA occlusion or maintained during and after CCA occlusion. For stroke models, we extended the bilateral CCA occlusion period from 1 to 2 hours, while the distal MCA remained occluded (Figure 3) [19]. The hypothermic duration at both temperatures was either 2 hours during or after CCA occlusion or 4 hours during and after CCA occlusion. We found that 2 hours of mild hypothermia (33°C) induced either during or after CCA occlusion did not confer protection [19]. This was unexpected because our previous study showed that 2 hours of intraischemic hypothermia (33°C) reduced infarct size in a 2-hour MCA suture occlusion model in rats [14]. In addition, as van der Worp et al. [29] reviewed, previous studies have reported a substantial reduction in infarction even at 35°C, when hypothermia commenced before or at the start of MCA occlusion, with protective effects that were not clearly time dependent. 

In our study, however, 4 hours of mild hypothermia applied during and after CCA release slightly, but significantly, reduced infarct size by 22%. When we further reduced hypothermia from 33°C to 30°C, 2 hours of moderate hypothermia during CCA occlusion increased protection, significantly reducing infarct size by 46% (Figure 3). Nevertheless, 2 additional hours of moderate hypothermia (4 hours total) did not offer additional protection, suggesting a limited effect of prolonged moderate hypothermia applied during and after CCA release [19]. 

Using confocal microscopy and Western blotting, we found that when intraischemic hypothermia reduced infarct size, the subcellular translocation of cytochrome c and apoptosis-inducing factor (AIF) was blocked in the ischemic penumbra. However, when hypothermia (either intraischemic or delayed mild hypothermia) did not reduce infarct size, no effect was observed on these proapoptotic factors [19]. This suggests that inhibition of cytochrome c and AIF release corresponded to the protective effect of hypothermia.

## 4. Limited Therapeutic Time Windows of Moderate Hypothermia (30°C) in a Focal Ischemia with Complete Reperfusion

**Figure 4:**
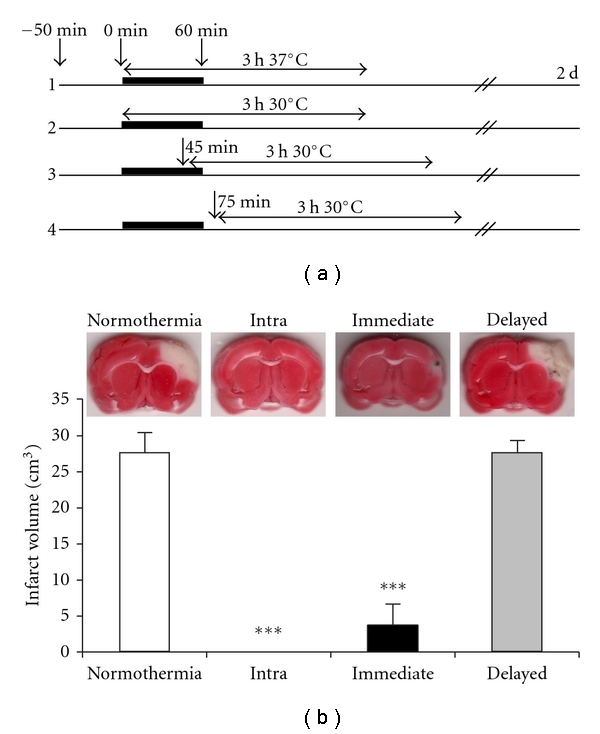
(revised from [18]) Limited therapeutic time windows for post-ischemic moderate hypothermia in a focal ischemia with complete reperfusion. (a) A diagram for experimental procedures comparing the protection of hypothermia. Rats were divided into 4 groups. Group 1, normothermia: body temperature was maintained at 37°C throughout the experiment. Group 2, intraischemic hypothermia: hypothermia was induced at ischemic onset and maintained for 3 h. Group 3, early hypothermia: body temperature was adjusted to 30°C 15 min before reperfusion and maintained for 3 h. Group 4, delayed hypothermia: body temperature was adjusted to 30°C 15 min after reperfusion and maintained for 3 h. (b) The upper panel shows representative infarcts stained by TTC. White areas are the infarct regions. The lower panel shows quantitation of infarct volumes. Values are mean ± S.E.M. (*n* = 8 per each group). ***P < 0.0001, versus normothermia.

After comparing the protective effects of both mild and moderate hypothermia in severe ischemic models with permanent distal MCA occlusion, we were not optimistic that mild hypothermia (33°C) could achieve protection. Thus, we focused on the therapeutic time window for moderate hypothermia (30°C) in a transient focal ischemic model with 1 hour of CCA and distal MCA occlusion, which allows complete reperfusion (Figure 4) [18]. Our aim was to determine the potential therapeutic time window for a brief moderate hypothermia in a less severe ischemic model. We found that 3 hours of moderate hypothermia started immediately after stroke onset spared almost all infarction (Figure 4(b)), and 3-hours of early moderate hypothermia induced 45 minutes after CCA occlusion markedly reduced infarction by more than 80%, whereas delayed hypothermia initiated 15 minutes after reperfusion did not prevent ischemic damage (Figure 4(b)) [18]. Together, these results suggest a very short therapeutic time window for a brief, moderate hypothermia. 

Our study on therapeutic time windows is limited by the short 3-hour duration of hypothermia. It is highly likely that the delayed onset of hypothermia would have been protective if prolonged hypothermia had been used. For instance, Colbourne et al. found that prolonged hypothermia (24 hours of 33°C plus 24 hours of 35°C) started 2.5 hours after the onset of ischemia robustly reduced infarct volume and attenuated behavior deficits in a focal ischemia model with a 90-minute MCA occlusion in rats [34]. Clark et al. reported that hypothermia (33°C) lasting 12, 24, or 48 hours was required to reduce infarct size and improve functional outcomes when hypothermia was instituted 1 hour after permanent distal MCA and CCA occlusion, and prolonged hypothermia (24 or 48 hours) was better than shorter hypothermia (12 hours) [35]. Furthermore, delayed hypothermia beginning 1 hour after ischemia appears to require prolonged periods (12 to 24 hours) to generate protection even for global ischemia lasting just 5 minutes [36]. Therefore, the limited therapeutic effects of post-ischemic hypothermia in our studies may be specific to the experimental settings in our laboratory.

Consistent with its protective effects, early hypothermia, but not delayed hypothermia, blocked TUNEL positive staining, a marker for apoptosis or cell death [18]. In addition, we found that early hypothermia attenuated the generation of superoxide compared with normothermia. However, both early and delayed hypothermia attenuated reductions in Mn-SOD protein levels and **δ**PKC cleavage in the ischemic penumbra, suggesting that both Mn-SOD and **δ**PKC cleavage may not be responsible for the differential protective effects of early and delayed hypothermia [18]. In addition, both early and delayed hypothermia preserved Akt phosphorylation. Nevertheless, only early hypothermia, but not delayed hypothermia, maintained PTEN phosphorylation (P-PTEN) [18], suggesting that P-PTEN may play a critical role in the protective effects of early hypothermia through the attenuation of ROS activity.

## 5. Discussion

As we have discussed, hypothermic studies performed in the laboratory have led to clinical investigations for cerebral ischemia. Significant enthusiasm for this approach still exists in the scientific community. A number of preliminary clinical trials (mostly phase I) to confirm the feasibility and safety of induced mild hypothermia for stroke patients have been completed, and several phase II clinical trials are currently in progress (http://clinicaltrials.gov/). However, whether mild-to-moderate hypothermia can be successfully translated clinically or, if successful, how long this will take has yet to be determined. 

The purpose of our basic research using animal models is to provide the rationale for clinical translation, although we cannot directly extrapolate settings from the laboratory to clinical trials. As discussed, our laboratory experiment is limited due to the short 3-hour duration of hypothermia, which contrasts to human clinical trials where hypothermia may last a few days. In addition, our study used infarct size as the criteria for evaluating the protective effects of hypothermia and not neurological function, as is often the case in clinical studies. Despite these limitations, our results serve as a warning of the persistent challenges we must confront as we seek to translate hypothermia to the clinic.

First of all, the most strikingly disappointing results from our studies are the limited protective effects of hypothermia, including mild hypothermia, and the short therapeutic time window of moderate hypothermia. If these observations are true, successful clinical translation of induced hypothermia may prove to be more difficult than anticipated to achieve. 

For example, we demonstrated that even intraischemic mild hypothermia (33°C) induced before ischemic onset failed to reduce infarct size in a focal ischemia model with permanent distal MCA occlusion and partial reperfusion upon bilateral CCA release. This model may be more severe than the model of MCA suture occlusion with reperfusion used by most laboratories, but we have no reason to believe it is more severe than strokes in humans. As previously discussed, many stroke patients suffer from permanent cerebral artery occlusion without reperfusion. To achieve protection, even our experimental ischemic models required reducing intraischemic hypothermia to 30°C or prolonging intraischemic mild hypothermia beyond CCA release. However, applying intraischemic hypothermia before stroke onset in clinical trials is nearly impossible, and inducing hypothermia in stroke patients beyond 33°C to 30°C is very difficult. Clinical trials often use mild rather than moderate hypothermia, and it takes significantly longer to reach the target temperature compared to experimental stroke in animal models.

Nevertheless, as we reviewed previously [6], other groups have shown that intraischemic mild hypothermia elicits protection even in permanent MCA occlusion models, in contrast to our recent studies. Our negative findings may simply reflect our specific setting and use of a unique model.

Second, the therapeutic time window for moderate hypothermia is extremely narrow after stroke onset, even in the 1-hour transient focal ischemic model. To achieve protection, 3 hours of moderate hypothermia must be induced as early as 45 minutes after stroke onset; a 30-minute delay rendered the moderate hypothermia ineffective. Again, it is highly unlikely that most stroke patients can receive hypothermic treatment within 1 hour of stroke onset. In most clinical studies, mild-to-moderate hypothermia was initiated as late as 5 to 6 hours after stroke, and one to several hours were required to reach target temperatures [37, 38]. In addition, patients may not have reperfusion, or if there is reperfusion, it may occur at a very late stage. 

Our studies on the underlying protective mechanisms may also offer some alternative clues or applications for clinical trials. For instance, we demonstrated that hypothermia reduces infarct size by preserving Akt activity and PTEN phosphorylation and by inhibiting ROS activity. If possible, pharmacological agents may be developed that improve Akt activity while inhibiting PTEN activity, or attenuating ROS production, and such pharmacological agents may be used in combination with induced hypothermia. 

In summary, despite confounding issues, laboratory studies have provided strong rationale for clinical application of hypothermia for acute stroke treatment. In clinical settings, a number of crucial variables need to be considered, including the onset time of hypothermia, its depth, and whether the strokes studied include reperfusion. Early reperfusion and rapid hypothermia initiation should be used to achieve maximal protection.
